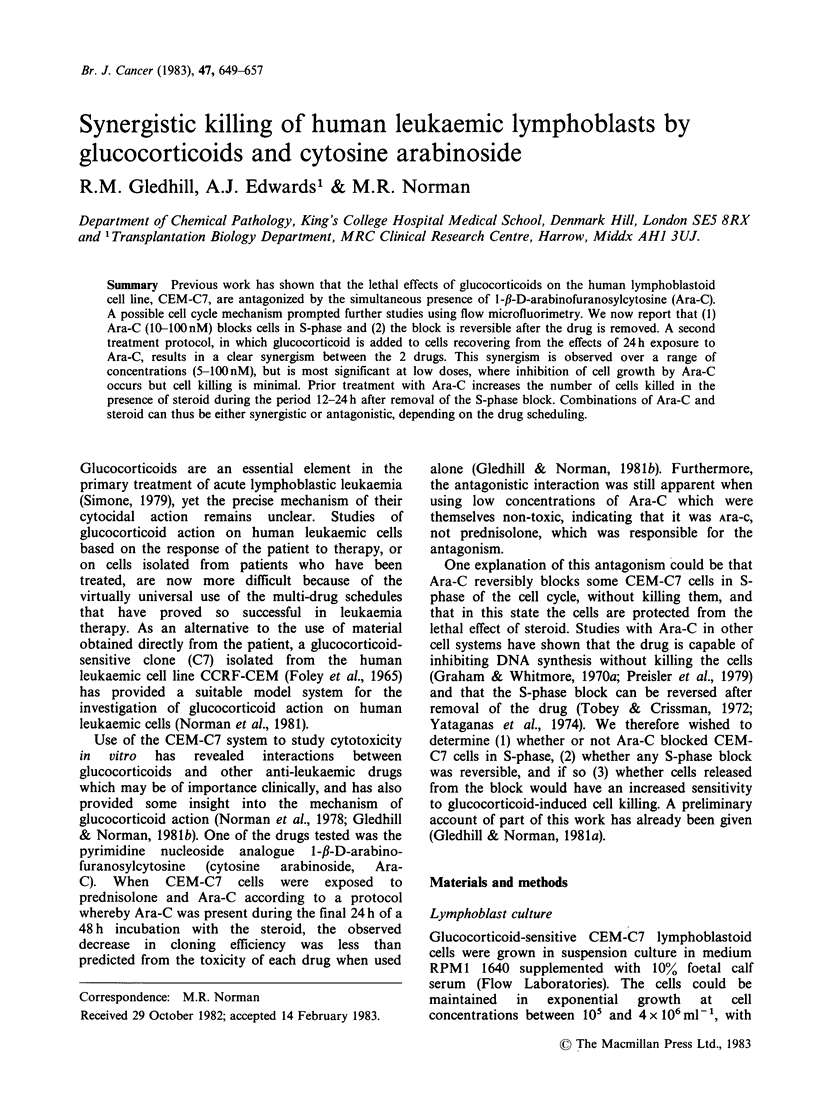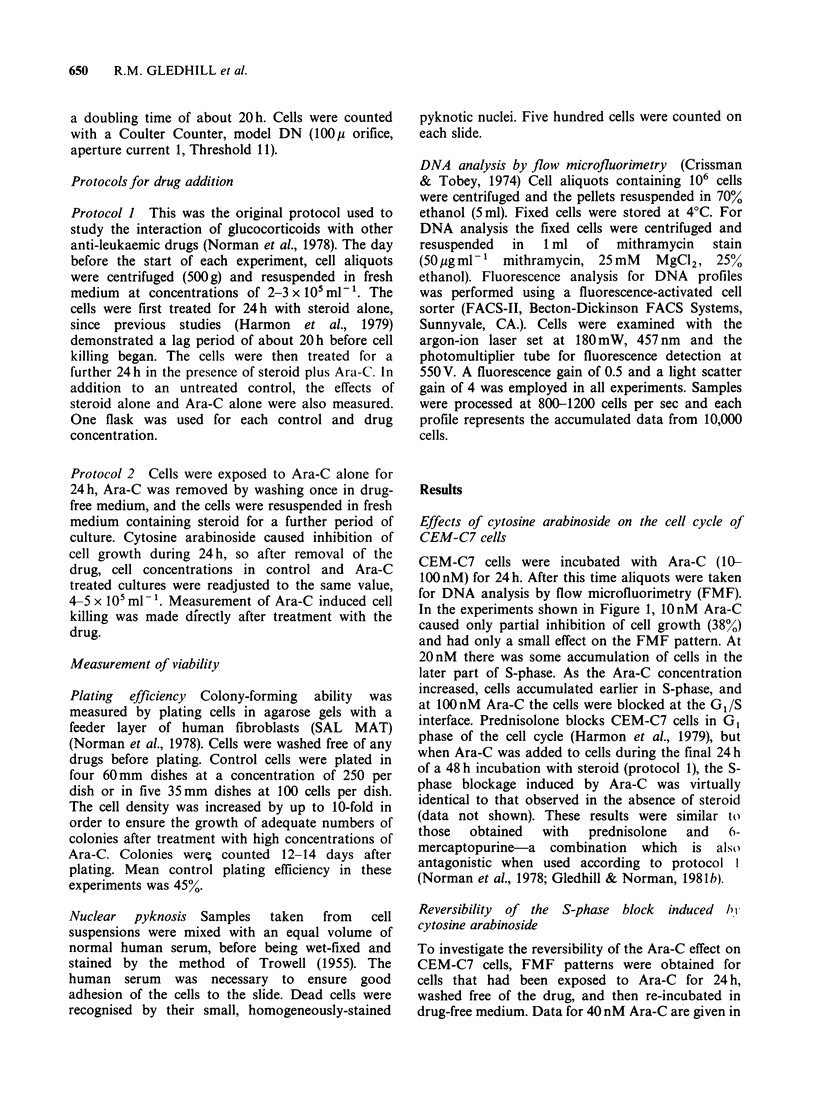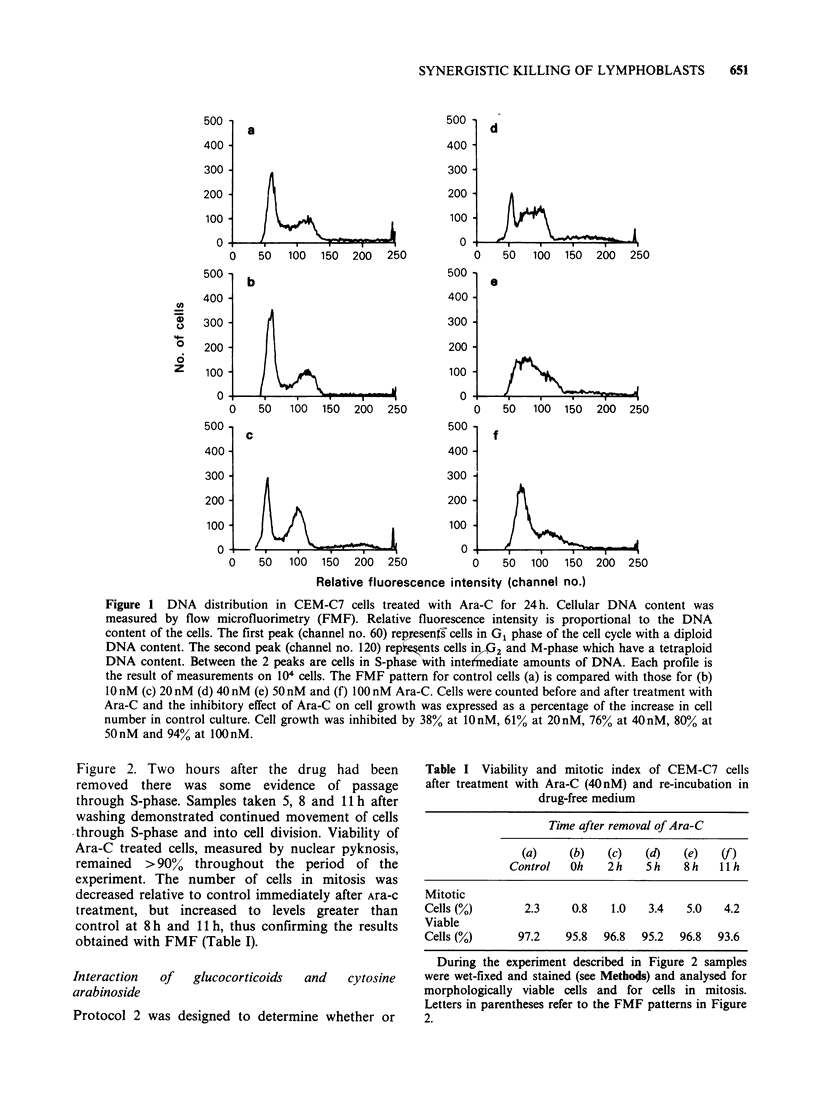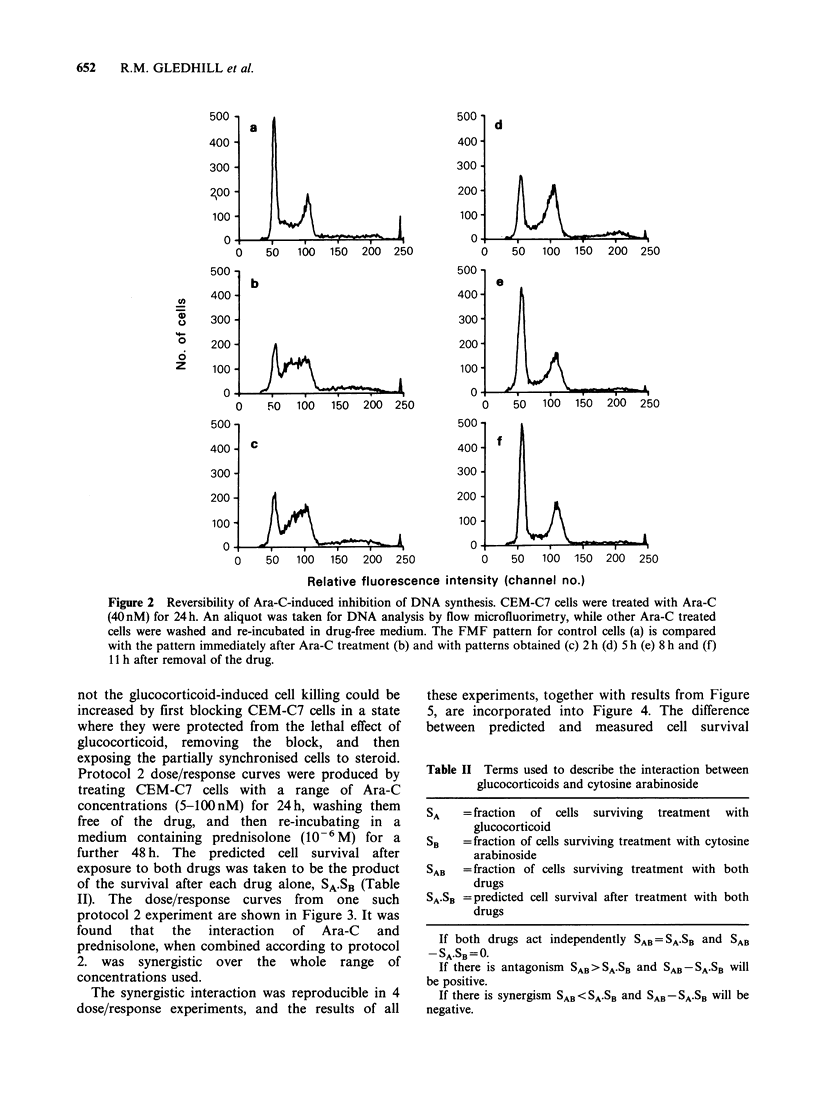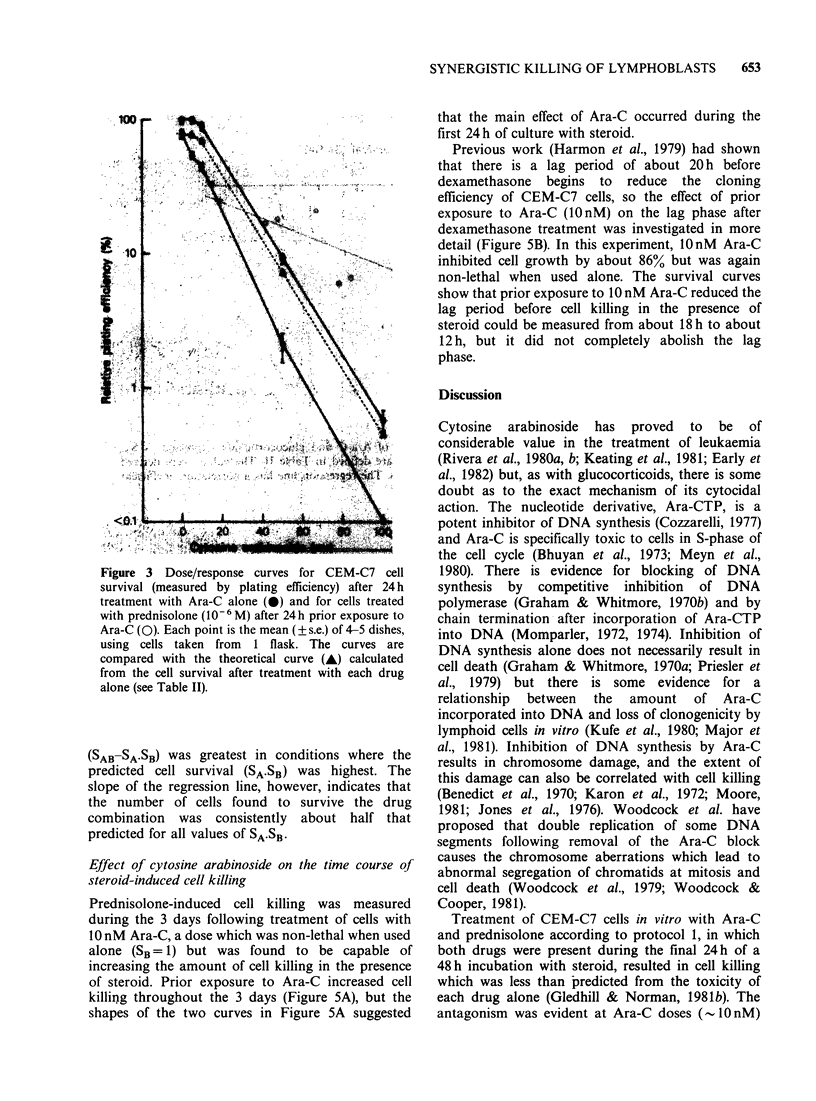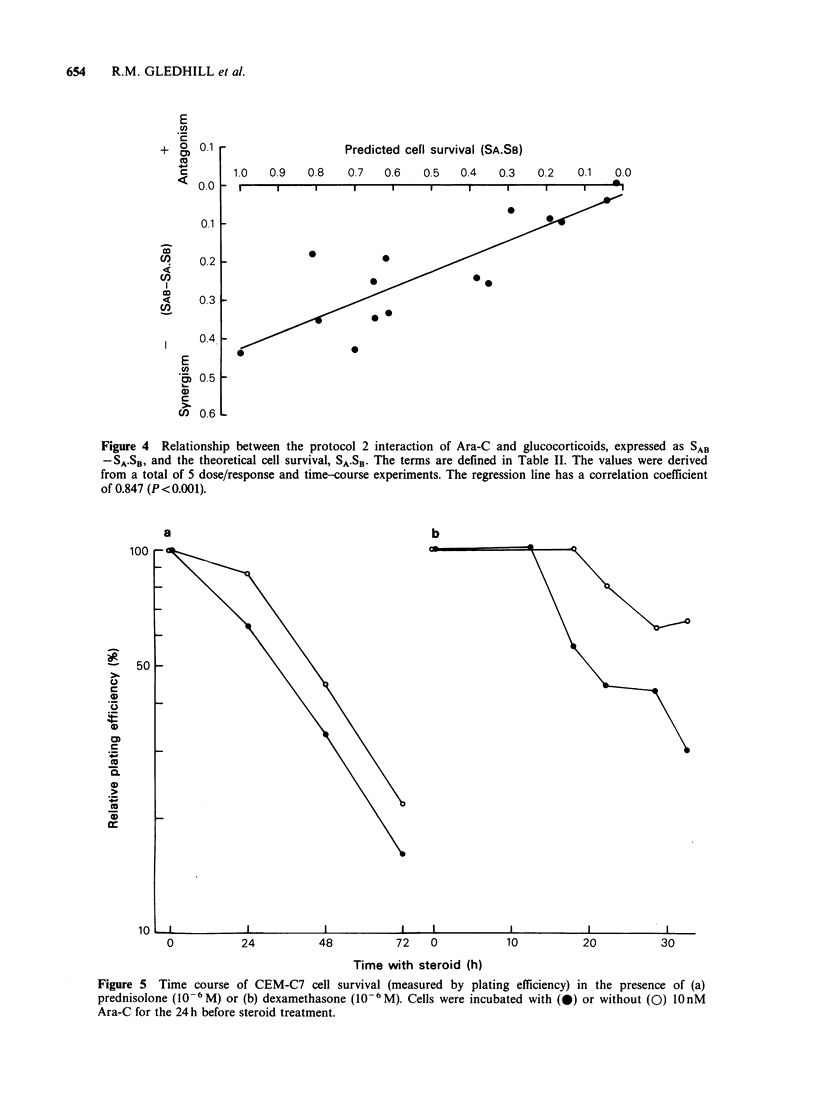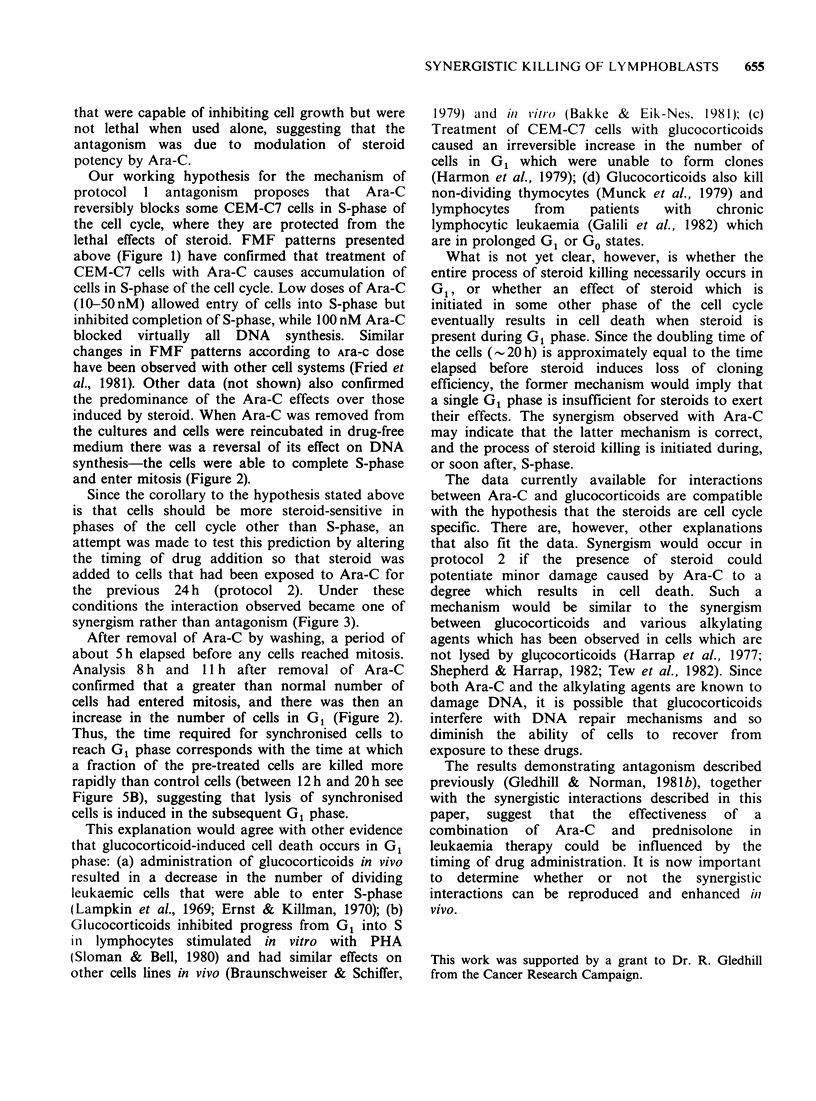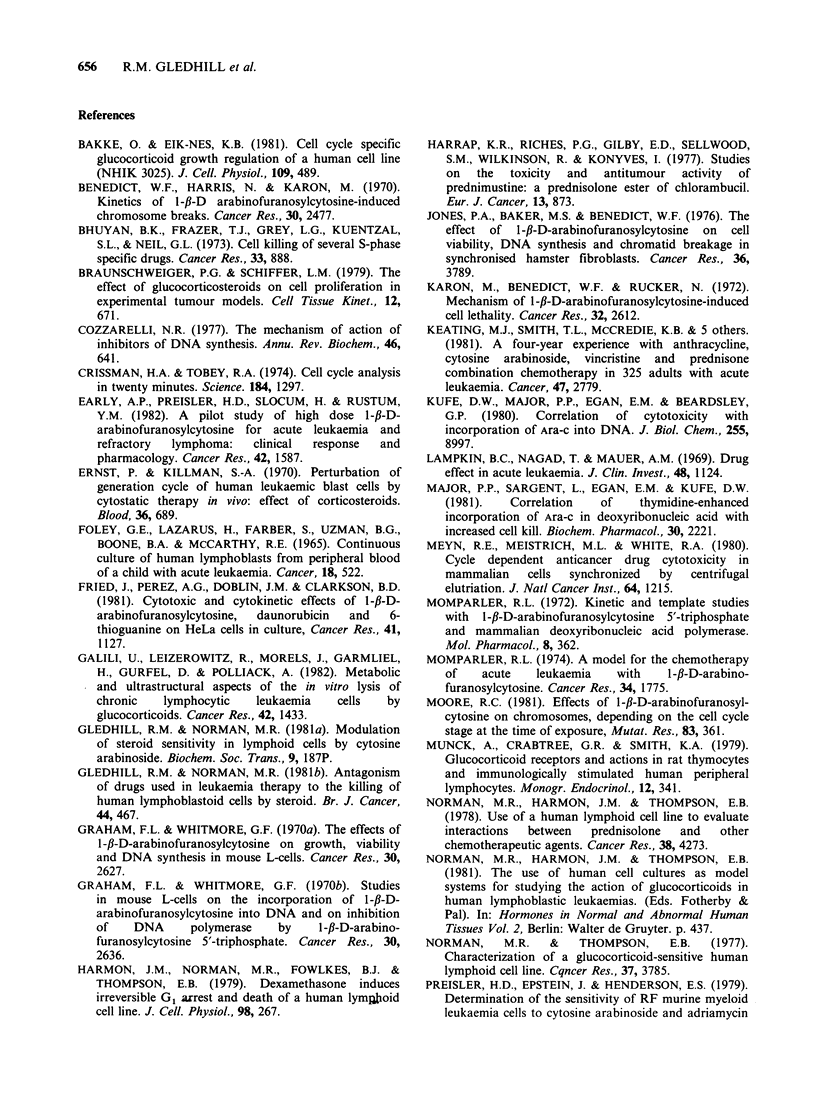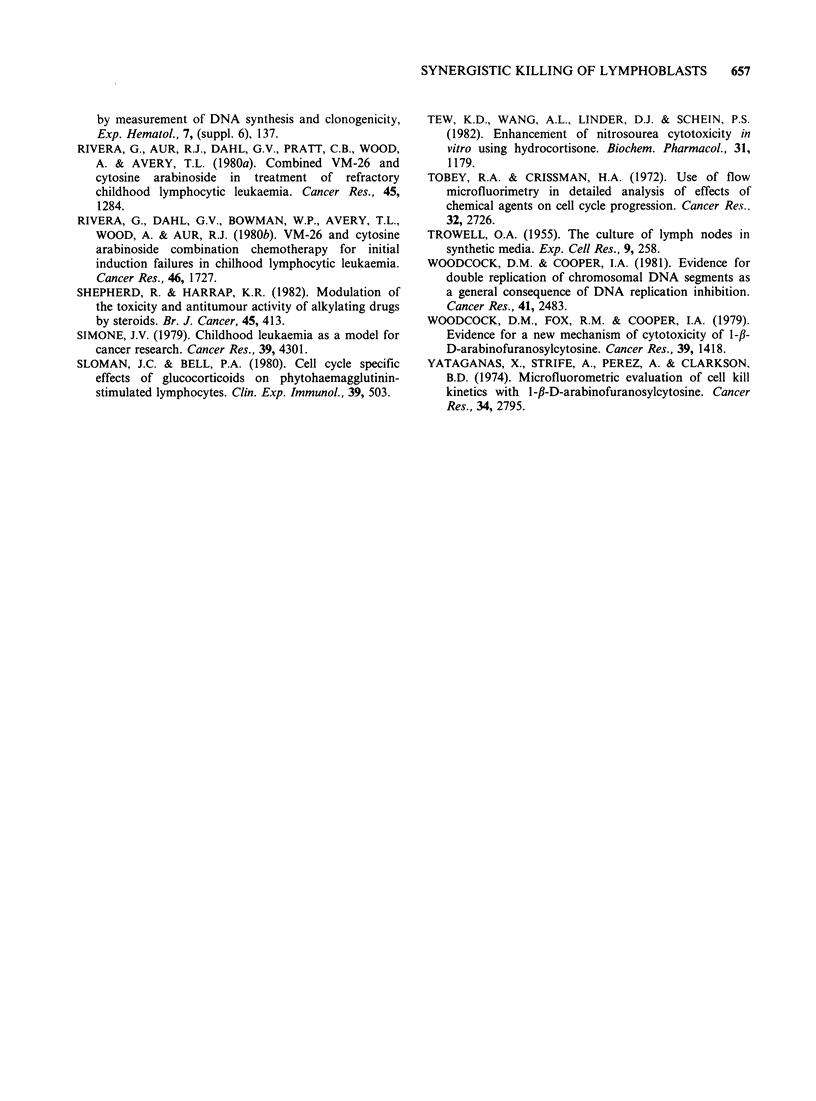# Synergistic killing of human leukaemic lymphoblasts by glucocorticoids and cytosine arabinoside.

**DOI:** 10.1038/bjc.1983.103

**Published:** 1983-05

**Authors:** R. M. Gledhill, A. J. Edwards, M. R. Norman

## Abstract

Previous work has shown that the lethal effects of glucocorticoids on the human lymphoblastoid cell line, CEM-C7, are antagonized by the simultaneous presence of 1-beta-D-arabinofuranosylcytosine (Ara-C). A possible cell cycle mechanism prompted further studies using flow microfluorimetry. We now report that (1) Ara-C (10-100 nM) blocks cells in S-phase and (2) the block is reversible after the drug is removed. A second treatment protocol, in which glucocorticoid is added to cells recovering from the effects of 24 h exposure to Ara-C, results in a clear synergism between the 2 drugs. This synergism is observed over a range of concentrations (5-100 nM), but is most significant at low doses, where inhibition of cell growth by Ara-C occurs but cell killing is minimal. Prior treatment with Ara-C increases the number of cells killed in the presence of steroid during the period 12-24 h after removal of the S-phase block. Combinations of Ara-C and steroid can thus be either synergistic or antagonistic, depending on the drug scheduling.


*Br. J. Cancer* (1983), **47**, 649–657

### Synergistic killing of human leukaemic lymphoblasts by glucocorticoids and cytosine arabinoside

R.M. Gledhill, A.J. Edwards[1] & M.R. Norman

*Department of Chemical Pathology, King's College Hospital Medical School, Denmark Hill, London SE5 8RX and [1]Transplantation Biology Department, MRC Clinical Research Centre, Harrow, Middx AH1 3UJ.*

**Summary** Previous work has shown that the lethal effects of glucocorticoids on the human lymphoblastoid cell line, CEM-C7, are antagonized by the simultaneous presence of 1-$\beta$-D-arabinofuranosylcytosine (Ara-C). A possible cell cycle mechanism prompted further studies using flow microfluorimetry. We now report that (1) Ara-C (10–100 nM) blocks cells in S-phase and (2) the block is reversible after the drug is removed. A second treatment protocol, in which glucocorticoid is added to cells recovering from the effects of 24 h exposure to Ara-C, results in a clear synergism between the 2 drugs. This synergism is observed over a range of concentrations (5–100 nM), but is most significant at low doses, where inhibition of cell growth by Ara-C occurs but cell killing is minimal. Prior treatment with Ara-C increases the number of cells killed in the presence of steroid during the period 12–24 h after removal of the S-phase block. Combinations of Ara-C and steroid can thus be either synergistic or antagonistic, depending on the drug scheduling.

Glucocorticoids are an essential element in the primary treatment of acute lymphoblastic leukaemia (Simone, 1979), yet the precise mechanism of their cytocidal action remains unclear. Studies of glucocorticoid action on human leukaemic cells based on the response of the patient to therapy, or on cells isolated from patients who have been treated, are now more difficult because of the virtually universal use of the multi-drug schedules that have proved so successful in leukaemia therapy. As an alternative to the use of material obtained directly from the patient, a glucocorticoid-sensitive clone (C7) isolated from the human leukaemic cell line CCRF-CEM (Foley *et al.*, 1965) has provided a suitable model system for the investigation of glucocorticoid action on human leukaemic cells (Norman *et al.*, 1981).

Use of the CEM-C7 system to study cytotoxicity *in vitro* has revealed interactions between glucocorticoids and other anti-leukaemic drugs which may be of importance clinically, and has also provided some insight into the mechanism of glucocorticoid action (Norman *et al.*, 1978; Gledhill & Norman, 1981*b*). One of the drugs tested was the pyrimidine nucleoside analogue 1-$\beta$-D-arabinofuranosylcytosine (cytosine arabinoside, Ara-C). When CEM-C7 cells were exposed to prednisolone and Ara-C according to a protocol whereby Ara-C was present during the final 24 h of a 48 h incubation with the steroid, the observed decrease in cloning efficiency was less than predicted from the toxicity of each drug when used alone (Gledhill & Norman, 1981*b*). Furthermore, the antagonistic interaction was still apparent when using low concentrations of Ara-C which were themselves non-toxic, indicating that it was Ara-C, not prednisolone, which was responsible for the antagonism.

One explanation of this antagonism could be that Ara-C reversibly blocks some CEM-C7 cells in S-phase of the cell cycle, without killing them, and that in this state the cells are protected from the lethal effect of steroid. Studies with Ara-C in other cell systems have shown that the drug is capable of inhibiting DNA synthesis without killing the cells (Graham & Whitmore, 1970*a*; Preisler *et al.*, 1979) and that the S-phase block can be reversed after removal of the drug (Tobey & Crissman, 1972; Yataganas *et al.*, 1974). We therefore wished to determine (1) whether or not Ara-C blocked CEM-C7 cells in S-phase, (2) whether any S-phase block was reversible, and if so (3) whether cells released from the block would have an increased sensitivity to glucocorticoid-induced cell killing. A preliminary account of part of this work has already been given (Gledhill & Norman, 1981*a*).

### Materials and methods

### *Lymphoblast culture*

Glucocorticoid-sensitive CEM-C7 lymphoblastoid cells were grown in suspension culture in medium RPM1 1640 supplemented with 10% foetal calf serum (Flow Laboratories). The cells could be maintained in exponential growth at cell concentrations between $10^5$ and $4 \times 10^6\,ml^{-1}$, with

Correspondence: M.R. Norman

Received 29 October 1982; accepted 14 February 1983.

© The Macmillan Press Ltd., 1983

a doubling time of about 20 h. Cells were counted with a Coulter Counter, model DN (100 μ orifice, aperture current 1, Threshold 11).

*Protocols for drug addition*

*Protocol 1* This was the original protocol used to study the interaction of glucocorticoids with other anti-leukaemic drugs (Norman *et al.*, 1978). The day before the start of each experiment, cell aliquots were centrifuged (500 g) and resuspended in fresh medium at concentrations of $2\text{–}3 \times 10^5\,ml^{-1}$. The cells were first treated for 24 h with steroid alone, since previous studies (Harmon *et al.*, 1979) demonstrated a lag period of about 20 h before cell killing began. The cells were then treated for a further 24 h in the presence of steroid plus Ara-C. In addition to an untreated control, the effects of steroid alone and Ara-C alone were also measured. One flask was used for each control and drug concentration.

*Protocol 2* Cells were exposed to Ara-C alone for 24 h, Ara-C was removed by washing once in drug-free medium, and the cells were resuspended in fresh medium containing steroid for a further period of culture. Cytosine arabinoside caused inhibition of cell growth during 24 h, so after removal of the drug, cell concentrations in control and Ara-C treated cultures were readjusted to the same value, $4\text{–}5 \times 10^5\,ml^{-1}$. Measurement of Ara-C induced cell killing was made directly after treatment with the drug.

*Measurement of viability*

*Plating efficiency* Colony-forming ability was measured by plating cells in agarose gels with a feeder layer of human fibroblasts (SAL MAT) (Norman *et al.*, 1978). Cells were washed free of any drugs before plating. Control cells were plated in four 60 mm dishes at a concentration of 250 per dish or in five 35 mm dishes at 100 cells per dish. The cell density was increased by up to 10-fold in order to ensure the growth of adequate numbers of colonies after treatment with high concentrations of Ara-C. Colonies were counted 12–14 days after plating. Mean control plating efficiency in these experiments was 45%.

*Nuclear pyknosis* Samples taken from cell suspensions were mixed with an equal volume of normal human serum, before being wet-fixed and stained by the method of Trowell (1955). The human serum was necessary to ensure good adhesion of the cells to the slide. Dead cells were recognised by their small, homogeneously-stained pyknotic nuclei. Five hundred cells were counted on each slide.

*DNA analysis by flow microfluorimetry* (Crissman & Tobey, 1974) Cell aliquots containing $10^6$ cells were centrifuged and the pellets resuspended in 70% ethanol (5 ml). Fixed cells were stored at 4°C. For DNA analysis the fixed cells were centrifuged and resuspended in 1 ml of mithramycin stain ($50\,\mu g\,ml^{-1}$ mithramycin, 25 mM $MgCl_2$, 25% ethanol). Fluorescence analysis for DNA profiles was performed using a fluorescence-activated cell sorter (FACS-II, Becton-Dickinson FACS Systems, Sunnyvale, CA.). Cells were examined with the argon-ion laser set at 180 mW, 457 nm and the photomultiplier tube for fluorescence detection at 550 V. A fluorescence gain of 0.5 and a light scatter gain of 4 was employed in all experiments. Samples were processed at 800–1200 cells per sec and each profile represents the accumulated data from 10,000 cells.

### Results

*Effects of cytosine arabinoside on the cell cycle of CEM-C7 cells*

CEM-C7 cells were incubated with Ara-C (10–100 nM) for 24 h. After this time aliquots were taken for DNA analysis by flow microfluorimetry (FMF). In the experiments shown in Figure 1, 10 nM Ara-C caused only partial inhibition of cell growth (38%) and had only a small effect on the FMF pattern. At 20 nM there was some accumulation of cells in the later part of S-phase. As the Ara-C concentration increased, cells accumulated earlier in S-phase, and at 100 nM Ara-C the cells were blocked at the $G_1/S$ interface. Prednisolone blocks CEM-C7 cells in $G_1$ phase of the cell cycle (Harmon *et al.*, 1979), but when Ara-C was added to cells during the final 24 h of a 48 h incubation with steroid (protocol 1), the S-phase blockage induced by Ara-C was virtually identical to that observed in the absence of steroid (data not shown). These results were similar to those obtained with prednisolone and 6-mercaptopurine—a combination which is also antagonistic when used according to protocol 1 (Norman *et al.*, 1978; Gledhill & Norman, 1981*b*).

*Reversibility of the S-phase block induced by cytosine arabinoside*

To investigate the reversibility of the Ara-C effect on CEM-C7 cells, FMF patterns were obtained for cells that had been exposed to Ara-C for 24 h, washed free of the drug, and then re-incubated in drug-free medium. Data for 40 nM Ara-C are given in

**Figure 1** DNA distribution in CEM-C7 cells treated with Ara-C for 24 h. Cellular DNA content was measured by flow microfluorimetry (FMF). Relative fluorescence intensity is proportional to the DNA content of the cells. The first peak (channel no. 60) represents cells in $G_1$ phase of the cell cycle with a diploid DNA content. The second peak (channel no. 120) represents cells in $G_2$ and M-phase which have a tetraploid DNA content. Between the 2 peaks are cells in S-phase with intermediate amounts of DNA. Each profile is the result of measurements on $10^4$ cells. The FMF pattern for control cells (a) is compared with those for (b) 10 nM (c) 20 nM (d) 40 nM (e) 50 nM and (f) 100 nM Ara-C. Cells were counted before and after treatment with Ara-C and the inhibitory effect of Ara-C on cell growth was expressed as a percentage of the increase in cell number in control culture. Cell growth was inhibited by 38% at 10 nM, 61% at 20 nM, 76% at 40 nM, 80% at 50 nM and 94% at 100 nM.

Figure 2. Two hours after the drug had been removed there was some evidence of passage through S-phase. Samples taken 5, 8 and 11 h after washing demonstrated continued movement of cells through S-phase and into cell division. Viability of Ara-C treated cells, measured by nuclear pyknosis, remained >90% throughout the period of the experiment. The number of cells in mitosis was decreased relative to control immediately after Ara-c treatment, but increased to levels greater than control at 8 h and 11 h, thus confirming the results obtained with FMF (Table I).

*Interaction of glucocorticoids and cytosine arabinoside*

Protocol 2 was designed to determine whether or

**Table I** Viability and mitotic index of CEM-C7 cells after treatment with Ara-C (40 nM) and re-incubation in drug-free medium

| | *Time after removal of Ara-C* | | | | | |
|---|---|---|---|---|---|---|
| | (*a*) *Control* | (*b*) 0*h* | (*c*) 2 *h* | (*d*) 5 *h* | (*e*) 8 *h* | (*f*) 11 *h* |
| Mitotic Cells (%) | 2.3 | 0.8 | 1.0 | 3.4 | 5.0 | 4.2 |
| Viable Cells (%) | 97.2 | 95.8 | 96.8 | 95.2 | 96.8 | 93.6 |

During the experiment described in Figure 2 samples were wet-fixed and stained (see **Methods**) and analysed for morphologically viable cells and for cells in mitosis. Letters in parentheses refer to the FMF patterns in Figure 2.

**Figure 2** Reversibility of Ara-C-induced inhibition of DNA synthesis. CEM-C7 cells were treated with Ara-C (40 nM) for 24 h. An aliquot was taken for DNA analysis by flow microfluorimetry, while other Ara-C treated cells were washed and re-incubated in drug-free medium. The FMF pattern for control cells (a) is compared with the pattern immediately after Ara-C treatment (b) and with patterns obtained (c) 2 h (d) 5 h (e) 8 h and (f) 11 h after removal of the drug.

not the glucocorticoid-induced cell killing could be increased by first blocking CEM-C7 cells in a state where they were protected from the lethal effect of glucocorticoid, removing the block, and then exposing the partially synchronised cells to steroid. Protocol 2 dose/response curves were produced by treating CEM-C7 cells with a range of Ara-C concentrations (5–100 nM) for 24 h, washing them free of the drug, and then re-incubating in a medium containing prednisolone ($10^{-6}$ M) for a further 48 h. The predicted cell survival after exposure to both drugs was taken to be the product of the survival after each drug alone, $S_A.S_B$ (Table II). The dose/response curves from one such protocol 2 experiment are shown in Figure 3. It was found that the interaction of Ara-C and prednisolone, when combined according to protocol 2. was synergistic over the whole range of concentrations used.

The synergistic interaction was reproducible in 4 dose/response experiments, and the results of all these experiments, together with results from Figure 5, are incorporated into Figure 4. The difference between predicted and measured cell survival

**Table II** Terms used to describe the interaction between glucocorticoids and cytosine arabinoside

| | |
|---|---|
| $S_A$ | =fraction of cells surviving treatment with glucocorticoid |
| $S_B$ | =fraction of cells surviving treatment with cytosine arabinoside |
| $S_{AB}$ | =fraction of cells surviving treatment with both drugs |
| $S_A.S_B$ | =predicted cell survival after treatment with both drugs |

If both drugs act independently $S_{AB}=S_A.S_B$ and $S_{AB}-S_A.S_B=0$.

If there is antagonism $S_{AB}>S_A.S_B$ and $S_{AB}-S_A.S_B$ will be positive.

If there is synergism $S_{AB}<S_A.S_B$ and $S_{AB}-S_A.S_B$ will be negative.

**Figure 3** Dose/response curves for CEM-C7 cell survival (measured by plating efficiency) after 24 h treatment with Ara-C alone (●) and for cells treated with prednisolone ($10^{-6}$ M) after 24 h prior exposure to Ara-C (○). Each point is the mean (±s.e.) of 4–5 dishes, using cells taken from 1 flask. The curves are compared with the theoretical curve (▲) calculated from the cell survival after treatment with each drug alone (see Table II).

($S_{AB}$–$S_A.S_B$) was greatest in conditions where the predicted cell survival ($S_A.S_B$) was highest. The slope of the regression line, however, indicates that the number of cells found to survive the drug combination was consistently about half that predicted for all values of $S_A.S_B$.

*Effect of cytosine arabinoside on the time course of steroid-induced cell killing*

Prednisolone-induced cell killing was measured during the 3 days following treatment of cells with 10 nM Ara-C, a dose which was non-lethal when used alone ($S_B=1$) but was found to be capable of increasing the amount of cell killing in the presence of steroid. Prior exposure to Ara-C increased cell killing throughout the 3 days (Figure 5A), but the shapes of the two curves in Figure 5A suggested that the main effect of Ara-C occurred during the first 24 h of culture with steroid.

Previous work (Harmon *et al.*, 1979) had shown that there is a lag period of about 20 h before dexamethasone begins to reduce the cloning efficiency of CEM-C7 cells, so the effect of prior exposure to Ara-C (10 nM) on the lag phase after dexamethasone treatment was investigated in more detail (Figure 5B). In this experiment, 10 nM Ara-C inhibited cell growth by about 86% but was again non-lethal when used alone. The survival curves show that prior exposure to 10 nM Ara-C reduced the lag period before cell killing in the presence of steroid could be measured from about 18 h to about 12 h, but it did not completely abolish the lag phase.

### Discussion

Cytosine arabinoside has proved to be of considerable value in the treatment of leukaemia (Rivera *et al.*, 1980*a*, *b*; Keating *et al.*, 1981; Early *et al.*, 1982) but, as with glucocorticoids, there is some doubt as to the exact mechanism of its cytocidal action. The nucleotide derivative, Ara-CTP, is a potent inhibitor of DNA synthesis (Cozzarelli, 1977) and Ara-C is specifically toxic to cells in S-phase of the cell cycle (Bhuyan *et al.*, 1973; Meyn *et al.*, 1980). There is evidence for blocking of DNA synthesis by competitive inhibition of DNA polymerase (Graham & Whitmore, 1970*b*) and by chain termination after incorporation of Ara-CTP into DNA (Momparler, 1972, 1974). Inhibition of DNA synthesis alone does not necessarily result in cell death (Graham & Whitmore, 1970*a*; Priesler *et al.*, 1979) but there is some evidence for a relationship between the amount of Ara-C incorporated into DNA and loss of clonogenicity by lymphoid cells *in vitro* (Kufe *et al.*, 1980; Major *et al.*, 1981). Inhibition of DNA synthesis by Ara-C results in chromosome damage, and the extent of this damage can also be correlated with cell killing (Benedict *et al.*, 1970; Karon *et al.*, 1972; Moore, 1981; Jones *et al.*, 1976). Woodcock *et al.* have proposed that double replication of some DNA segments following removal of the Ara-C block causes the chromosome aberrations which lead to abnormal segregation of chromatids at mitosis and cell death (Woodcock *et al.*, 1979; Woodcock & Cooper, 1981).

Treatment of CEM-C7 cells *in vitro* with Ara-C and prednisolone according to protocol 1, in which both drugs were present during the final 24 h of a 48 h incubation with steroid, resulted in cell killing which was less than predicted from the toxicity of each drug alone (Gledhill & Norman, 1981*b*). The antagonism was evident at Ara-C doses (~10 nM)

**Figure 4** Relationship between the protocol 2 interaction of Ara-C and glucocorticoids, expressed as $S_{AB} - S_A.S_B$, and the theoretical cell survival, $S_A.S_B$. The terms are defined in Table II. The values were derived from a total of 5 dose/response and time-course experiments. The regression line has a correlation coefficient of 0.847 ($P<0.001$).

**Figure 5** Time course of CEM-C7 cell survival (measured by plating efficiency) in the presence of (a) prednisolone ($10^{-6}$ M) or (b) dexamethasone ($10^{-6}$ M). Cells were incubated with (●) or without (○) 10 nM Ara-C for the 24 h before steroid treatment.

that were capable of inhibiting cell growth but were not lethal when used alone, suggesting that the antagonism was due to modulation of steroid potency by Ara-C.

Our working hypothesis for the mechanism of protocol 1 antagonism proposes that Ara-C reversibly blocks some CEM-C7 cells in S-phase of the cell cycle, where they are protected from the lethal effects of steroid. FMF patterns presented above (Figure 1) have confirmed that treatment of CEM-C7 cells with Ara-C causes accumulation of cells in S-phase of the cell cycle. Low doses of Ara-C (10–50 nM) allowed entry of cells into S-phase but inhibited completion of S-phase, while 100 nM Ara-C blocked virtually all DNA synthesis. Similar changes in FMF patterns according to ara-c dose have been observed with other cell systems (Fried *et al.*, 1981). Other data (not shown) also confirmed the predominance of the Ara-C effects over those induced by steroid. When Ara-C was removed from the cultures and cells were reincubated in drug-free medium there was a reversal of its effect on DNA synthesis—the cells were able to complete S-phase and enter mitosis (Figure 2).

Since the corollary to the hypothesis stated above is that cells should be more steroid-sensitive in phases of the cell cycle other than S-phase, an attempt was made to test this prediction by altering the timing of drug addition so that steroid was added to cells that had been exposed to Ara-C for the previous 24 h (protocol 2). Under these conditions the interaction observed became one of synergism rather than antagonism (Figure 3).

After removal of Ara-C by washing, a period of about 5 h elapsed before any cells reached mitosis. Analysis 8 h and 11 h after removal of Ara-C confirmed that a greater than normal number of cells had entered mitosis, and there was then an increase in the number of cells in $G_1$ (Figure 2). Thus, the time required for synchronised cells to reach $G_1$ phase corresponds with the time at which a fraction of the pre-treated cells are killed more rapidly than control cells (between 12 h and 20 h see Figure 5B), suggesting that lysis of synchronised cells is induced in the subsequent $G_1$ phase.

This explanation would agree with other evidence that glucocorticoid-induced cell death occurs in $G_1$ phase: (a) administration of glucocorticoids *in vivo* resulted in a decrease in the number of dividing leukaemic cells that were able to enter S-phase (Lampkin *et al.*, 1969; Ernst & Killman, 1970); (b) Glucocorticoids inhibited progress from $G_1$ into S in lymphocytes stimulated *in vitro* with PHA (Sloman & Bell, 1980) and had similar effects on other cells lines *in vivo* (Braunschweiser & Schiffer, 1979) and *in vitro* (Bakke & Eik-Nes, 1981); (c) Treatment of CEM-C7 cells with glucocorticoids caused an irreversible increase in the number of cells in $G_1$ which were unable to form clones (Harmon *et al.*, 1979); (d) Glucocorticoids also kill non-dividing thymocytes (Munck *et al.*, 1979) and lymphocytes from patients with chronic lymphocytic leukaemia (Galili *et al.*, 1982) which are in prolonged $G_1$ or $G_0$ states.

What is not yet clear, however, is whether the entire process of steroid killing necessarily occurs in $G_1$, or whether an effect of steroid which is initiated in some other phase of the cell cycle eventually results in cell death when steroid is present during $G_1$ phase. Since the doubling time of the cells ($\sim$20 h) is approximately equal to the time elapsed before steroid induces loss of cloning efficiency, the former mechanism would imply that a single $G_1$ phase is insufficient for steroids to exert their effects. The synergism observed with Ara-C may indicate that the latter mechanism is correct, and the process of steroid killing is initiated during, or soon after, S-phase.

The data currently available for interactions between Ara-C and glucocorticoids are compatible with the hypothesis that the steroids are cell cycle specific. There are, however, other explanations that also fit the data. Synergism would occur in protocol 2 if the presence of steroid could potentiate minor damage caused by Ara-C to a degree which results in cell death. Such a mechanism would be similar to the synergism between glucocorticoids and various alkylating agents which has been observed in cells which are not lysed by glucocorticoids (Harrap *et al.*, 1977; Shepherd & Harrap, 1982; Tew *et al.*, 1982). Since both Ara-C and the alkylating agents are known to damage DNA, it is possible that glucocorticoids interfere with DNA repair mechanisms and so diminish the ability of cells to recover from exposure to these drugs.

The results demonstrating antagonism described previously (Gledhill & Norman, 1981*b*), together with the synergistic interactions described in this paper, suggest that the effectiveness of a combination of Ara-C and prednisolone in leukaemia therapy could be influenced by the timing of drug administration. It is now important to determine whether or not the synergistic interactions can be reproduced and enhanced *in vivo*.

This work was supported by a grant to Dr. R. Gledhill from the Cancer Research Campaign.

### References

BAKKE, O. & EIK-NES, K.B. (1981). Cell cycle specific glucocorticoid growth regulation of a human cell line (NHIK 3025). *J. Cell. Physiol.*, **109**, 489.

BENEDICT, W.F., HARRIS, N. & KARON, M. (1970). Kinetics of 1-β-D arabinofuranosylcytosine-induced chromosome breaks. *Cancer Res.*, **30**, 2477.

BHUYAN, B.K., FRAZER, T.J., GREY, L.G., KUENTZAL, S.L., & NEIL, G.L. (1973). Cell killing of several S-phase specific drugs. *Cancer Res.*, **33**, 888.

BRAUNSCHWEIGER, P.G. & SCHIFFER, L.M. (1979). The effect of glucocorticosteroids on cell proliferation in experimental tumour models. *Cell Tissue Kinet.*, **12**, 671.

COZZARELLI, N.R. (1977). The mechanism of action of inhibitors of DNA synthesis. *Annu. Rev. Biochem.*, **46**, 641.

CRISSMAN, H.A. & TOBEY, R.A. (1974). Cell cycle analysis in twenty minutes. *Science*. **184**, 1297.

EARLY, A.P., PREISLER, H.D., SLOCUM, H. & RUSTUM, Y.M. (1982). A pilot study of high dose 1-β-D-arabinofuranosylcytosine for acute leukaemia and refractory lymphoma: clinical response and pharmacology. *Cancer Res.*, **42**, 1587.

ERNST, P. & KILLMAN, S.-A. (1970). Perturbation of generation cycle of human leukaemic blast cells by cytostatic therapy *in vivo*: effect of corticosteroids. *Blood*, **36**, 689.

FOLEY, G.E., LAZARUS, H., FARBER, S., UZMAN, B.G., BOONE, B.A. & MCCARTHY, R.E. (1965). Continuous culture of human lymphoblasts from peripheral blood of a child with acute leukaemia. *Cancer*, **18**, 522.

FRIED, J., PEREZ, A.G., DOBLIN, J.M. & CLARKSON, B.D. (1981). Cytotoxic and cytokinetic effects of 1-β-D-arabinofuranosylcytosine, daunorubicin and 6-thioguanine on HeLa cells in culture, *Cancer Res.*, **41**, 1127.

GALILI, U., LEIZEROWITZ, R., MORELS, J., GARMLIEL, H., GURFEL, D. & POLLIACK, A. (1982). Metabolic and ultrastructural aspects of the *in vitro* lysis of chronic lymphocytic leukaemia cells by glucocorticoids. *Cancer Res.*, **42**, 1433.

GLEDHILL, R.M. & NORMAN, M.R. (1981*a*). Modulation of steroid sensitivity in lymphoid cells by cytosine arabinoside. *Biochem. Soc. Trans.*, **9**, 187P.

GLEDHILL, R.M. & NORMAN, M.R. (1981*b*). Antagonism of drugs used in leukaemia therapy to the killing of human lymphoblastoid cells by steroid. *Br. J. Cancer*, **44**, 467.

GRAHAM, F.L. & WHITMORE, G.F. (1970*a*). The effects of 1-β-D-arabinofuranosylcytosine on growth, viability and DNA synthesis in mouse L-cells. *Cancer Res.*, **30**, 2627.

GRAHAM, F.L. & WHITMORE, G.F. (1970*b*). Studies in mouse L-cells on the incorporation of 1-β-D-arabinofuranosylcytosine into DNA and on inhibition of DNA polymerase by 1-β-D-arabinofuranosylcytosine 5′-triphosphate. *Cancer Res.*, **30**, 2636.

HARMON, J.M., NORMAN, M.R., FOWLKES, B.J. & THOMPSON, E.B. (1979). Dexamethasone induces irreversible $G_1$ arrest and death of a human lymphoid cell line. *J. Cell. Physiol.*, **98**, 267.

HARRAP, K.R., RICHES, P.G., GILBY, E.D., SELLWOOD, S.M., WILKINSON, R. & KONYVES, I. (1977). Studies on the toxicity and antitumour activity of prednimustine: a prednisolone ester of chlorambucil. *Eur. J. Cancer*, **13**, 873.

JONES, P.A., BAKER, M.S. & BENEDICT, W.F. (1976). The effect of 1-β-D-arabinofuranosylcytosine on cell viability, DNA synthesis and chromatid breakage in synchronised hamster fibroblasts. *Cancer Res.*, **36**, 3789.

KARON, M., BENEDICT, W.F. & RUCKER, N. (1972). Mechanism of 1-β-D-arabinofuranosylcytosine-induced cell lethality. *Cancer Res.*, **32**, 2612.

KEATING, M.J., SMITH, T.L., MCCREDIE, K.B. & 5 others. (1981). A four-year experience with anthracycline, cytosine arabinoside, vincristine and prednisone combination chemotherapy in 325 adults with acute leukaemia. *Cancer*, **47**, 2779.

KUFE, D.W., MAJOR, P.P., EGAN, E.M. & BEARDSLEY, G.P. (1980). Correlation of cytotoxicity with incorporation of ARA-C into DNA. *J. Biol. Chem.*, **255**, 8997.

LAMPKIN, B.C., NAGAD, T. & MAUER, A.M. (1969). Drug effect in acute leukaemia. *J. Clin. Invest.*, **48**, 1124.

MAJOR, P.P., SARGENT, L., EGAN, E.M. & KUFE, D.W. (1981). Correlation of thymidine-enhanced incorporation of ARA-C in deoxyribonucleic acid with increased cell kill. *Biochem. Pharmacol.*, **30**, 2221.

MEYN, R.E., MEISTRICH, M.L. & WHITE, R.A. (1980). Cycle dependent anticancer drug cytotoxicity in mammalian cells synchronized by centrifugal elutriation. *J. Natl Cancer Inst.*, **64**, 1215.

MOMPARLER, R.L. (1972). Kinetic and template studies with 1-β-D-arabinofuranosylcytosine 5′-triphosphate and mammalian deoxyribonucleic acid polymerase. *Mol. Pharmacol.*, **8**, 362.

MOMPARLER, R.L. (1974). A model for the chemotherapy of acute leukaemia with 1-β-D-arabinofuranosylcytosine. *Cancer Res.*, **34**, 1775.

MOORE, R.C. (1981). Effects of 1-β-D-arabinofuranosylcytosine on chromosomes, depending on the cell cycle stage at the time of exposure, *Mutat. Res.*, **83**, 361.

MUNCK, A., CRABTREE, G.R. & SMITH, K.A. (1979). Glucocorticoid receptors and actions in rat thymocytes and immunologically stimulated human peripheral lymphocytes. *Monogr. Endocrinol.*, **12**, 341.

NORMAN, M.R., HARMON, J.M. & THOMPSON, E.B. (1978). Use of a human lymphoid cell line to evaluate interactions between prednisolone and other chemotherapeutic agents. *Cancer Res.*, **38**, 4273.

NORMAN, M.R., HARMON, J.M. & THOMPSON, E.B. (1981). The use of human cell cultures as model systems for studying the action of glucocorticoids in human lymphoblastic leukaemias. (Eds. Fotherby & Pal). In: *Hormones in Normal and Abnormal Human Tissues Vol. 2*, Berlin: Walter de Gruyter. p. 437.

NORMAN, M.R. & THOMPSON, E.B. (1977). Characterization of a glucocorticoid-sensitive human lymphoid cell line. *Cqncer Res.*, **37**, 3785.

PREISLER, H.D., EPSTEIN, J. & HENDERSON, E.S. (1979). Determination of the sensitivity of RF murine myeloid leukaemia cells to cytosine arabinoside and adriamycin by measurement of DNA synthesis and clonogenicity, *Exp. Hematol.*, **7**, (suppl. 6), 137.

RIVERA, G., AUR, R.J., DAHL, G.V., PRATT, C.B., WOOD, A. & AVERY, T.L. (1980*a*). Combined VM-26 and cytosine arabinoside in treatment of refractory childhood lymphocytic leukaemia. *Cancer Res.*, **45**, 1284.

RIVERA, G., DAHL, G.V., BOWMAN, W.P., AVERY, T.L., WOOD, A. & AUR, R.J. (1980*b*). VM-26 and cytosine arabinoside combination chemotherapy for initial induction failures in chilhood lymphocytic leukaemia. *Cancer Res.*, **46**, 1727.

SHEPHERD, R. & HARRAP, K.R. (1982). Modulation of the toxicity and antitumour activity of alkylating drugs by steroids. *Br. J. Cancer*, **45**, 413.

SIMONE, J.V. (1979). Childhood leukaemia as a model for cancer research. *Cancer Res.*, **39**, 4301.

SLOMAN, J.C. & BELL, P.A. (1980). Cell cycle specific effects of glucocorticoids on phytohaemagglutinin-stimulated lymphocytes. *Clin. Exp. Immunol.*, **39**, 503.

TEW, K.D., WANG, A.L., LINDER, D.J. & SCHEIN, P.S. (1982). Enhancement of nitrosourea cytotoxicity *in vitro* using hydrocortisone. *Biochem. Pharmacol.*, **31**, 1179.

TOBEY, R.A. & CRISSMAN, H.A. (1972). Use of flow microfluorimetry in detailed analysis of effects of chemical agents on cell cycle progression. *Cancer Res.*, **32**, 2726.

TROWELL, O.A. (1955). The culture of lymph nodes in synthetic media. *Exp. Cell Res.*, **9**, 258.

WOODCOCK, D.M. & COOPER, I.A. (1981). Evidence for double replication of chromosomal DNA segments as a general consequence of DNA replication inhibition. *Cancer Res.*, **41**, 2483.

WOODCOCK, D.M., FOX, R.M. & COOPER, I.A. (1979). Evidence for a new mechanism of cytotoxicity of 1-$\beta$-D-arabinofuranosylcytosine. *Cancer Res.*, **39**, 1418.

YATAGANAS, X., STRIFE, A., PEREZ, A. & CLARKSON, B.D. (1974). Microfluorometric evaluation of cell kill kinetics with 1-$\beta$-D-arabinofuranosylcytosine. *Cancer Res.*, **34**, 2795.